# Dnmt1 is required for the development of auditory organs via cell cycle arrest and Fgf signalling

**DOI:** 10.1111/cpr.13225

**Published:** 2022-03-29

**Authors:** Dongmei Tang, Shimei Zheng, Zhiwei Zheng, Chang Liu, Jiner Zhang, Renchun Yan, Cheng Wu, Na Zuo, Lijuan Wu, Hongfei Xu, Shaofeng Liu, Yingzi He

**Affiliations:** ^1^ ENT Institute and Otorhinolaryngology Department, Eye and ENT Hospital, NHC Key Laboratory of Hearing Medicine Research Fudan University Shanghai China; ^2^ Department of Otolaryngology‐Head and Neck Surgery Yijishan Hospital of Wannan Medical College Wuhu China; ^3^ Department of Forensic Medicine Soochow University Suzhou China

## Abstract

**Objectives:**

To explore the role of DNA methyltransferase 1 (DNMT1) in the development of auditory system using zebrafish as experimental model.

**Methods:**

Morpholino oligonucleotide was used to induce Dnmt1 deficiency. RNA sequencing, in situ hybridization (ISH), whole genomic bisulfide sequencing (WGBS) and immunostaining were used to investigate the morphologic alterations and mechanisms.

**Results:**

We found that downregulation of Dnmt1 induced decreased number of neuromasts and repressed cell proliferation of primordium in the developing posterior lateral line system of zebrafish. The ISH data uncovered that Fgf signalling pathway was inhibited and the expression of chemokine members *cxcr4b*, *cxcr7b* and *cxcl12a* were interfered, while *lef1* expression was increased after inhibiting Dnmt1. Additionally, Dnmt1 downregulation led to malformed otoliths and deformed semicircular canals, and hair cell differentiation in utricle and saccule was inhibited severely. The in situ staining of otic placode markers *pax2/5* and *fgf 3/8/10* was decreased when Dnmt1 downregulated. The WGBS analysis demonstrated that the global methylation status was markedly downregulated, and cell cycle genes were among those most differently expressed between Dnmt1 morphants and the controls. Further ISH analysis confirmed the findings by RNA‐seq and WGBS assay that *cdkn1a* and *tp53* were both upregulated after knockdown of Dnmt1.

**Conclusion:**

Our results revealed that Dnmt1 is essential for the development of zebrafish auditory organ through regulating cell cycle genes together with Wnt and Fgf signalling pathways.

## Graphical abstract

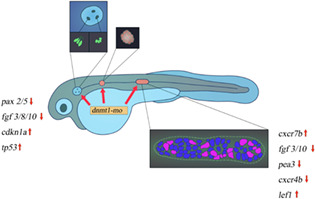

## 1 INTRODUCTION

Hearing disorders, often related to inner ear agenesis or injury, are serious health issues that affect quality of life. Thus, detecting the pivotal genes during auditory organogenesis is crucial to explore the potential strategy for hearing impairment. The zebrafish model has been increasingly applied in understanding the molecular genetic principle of inner ear dysplasia and related diseases.^1^, ^2^ The lateral line system is the sensory organ of zebrafish that detects water movements and sound, which is composed of the anterior lateral line in the head region and the posterior lateral line (PLL) behind the inner ear.^3^ The PLL primordium is a cluster of 100–150 cells that have the ability to migrate caudally to the end of the embryo, periodically deposit neuromasts at regular intervals, and finally form five or six truncal neuromasts and two or three terminal neuromasts.^4^ The mature neuromasts are composed of hair cells (HCs) and surrounding supporting cells, which are structurally and functionally similar with mammalian inner ear.^5^, ^6^The inner ear of zebrafish is composed of three pairs of semicircular canals (anterior, posterior and lateral) and two pairs of otolith organs named utricle and saccule.^6^, ^7^ The utricular macula has vestibular (balance) function, while the saccular macula has a primarily auditory (hearing) function.^8^ The formation of inner ear is a complex process, involving multiple factors and multistep actions.^6^ In addition to the genetic mechanism, there are also epigenetic mechanisms in the development of inner ear.^9^, ^10^

Epigenetics is on the study of heritable and reversible changes in gene expression patterns rather than altering the intrinsic DNA sequence. DNA methylation is one of the major forms of epigenetic modifications, which is catalysed by DNA methyltransferases family (DNMTs) through adding a methyl group to CpG dinucleotides at the fifth carbon of cytosine residue.^11^ The human canonical DNMTs family is composed of DNMT1, DNMT3A and DNMT3B.^12^ DNMT1 prefers to methylate hemi‐methylated DNA, while DNMT3A and DNMT3B can catalyse a naked or unmodified DNA as well as hemi‐methylated DNA, thus they are so‐called the maintenance methyltransferase (DNMT1) and the de novo methyltransferases (DNMT3A/3B).^13^ In zebrafish genome, there are eight DNA methyltransferases, among which Dnmt3, 4, 5 and 7 have high homology with human and mouse DNMT3B, while Dnmt6 and 8 in zebrafish and DNMT3A in mammals are high homologous.^14^ Similar with the mammals, Dnmt1 is also the maintenance enzyme in zebrafish.^15^

*Dnmt*(−/−) homozygous mice between E8.0 and E10.5 can result in a lethal embryo, suggesting a requirement of DNA methylation in embryonic development.^16^ Alterations in DNA methylation at specific CpG sites have been recently identified as a predictor of ageing and are used to estimate animal age in mammals and vertebrates like zebrafish.^17^ DNMT1 has been widely reported to play pivotal roles in a variety of complex life phenomena, including organ development, ageing, tumorigenesis and other diseases.^18^, ^19^ In Dnmt1 conditional knockout mice (Dnmt1[Δalb]) model, hepatocyte senescence is promoted and cell proliferation is inhibited, eventually causing cell death.^20^ Additionally, the methylation of genomic DNA is found active in different types of malignant cells, indicating an indispensable functionality of DNA methylation in cancer.^21^ For example, in osteosarcoma, decreased expression of DNMT1 is detected after MiR‐139‐5p overexpression, which induces the suppression of cell growth, migration and invasion.^22^ Low expression of *Dnmt1* also triggers structural and functional disorders of other tissues, that DNA hypomethylation has been found responsible for the dysregulation or even the lethality of GI smooth muscle cells.^23^ In a recent report, DNMT1 mutations have been identified in four kindreds with hereditary sensory and autonomic neuropathy (HSAN1), characterized by dementia and hearing loss,^24^ but the precise function of DNMT1 in auditory organs remains uncovered.

In this study, we detected a spatio‐temporal specific expression pattern of Dnmt1 during early embryonic development of zebrafish by whole mount in situ hybridization (WISH). The strong expression pattern in the eye, inner ear and lateral line system suggests that Dnmt1 is vital in the development and differentiation of these organs. We downregulated the expression of Dnmt1 by the method of microinjection with antisense morpholinos and the results demonstrated that Dnmt1‐MO embryos showed a certain degree of development delay and deformities including abnormal otolith organs, malformed semicircular canals and decreased number of neuromasts in lateral line system compared with the control group. Furthermore, we investigated that Dnmt1 modulated the development of auditory organs of zebrafish by regulating otic vesicle (OV) characterized genes, FGF signalling and cell cycle signalling pathways through ISH, RNA‐seq and WGBS analysis. Our research helps to deepen the understanding of the regulation mechanism of inner ear development.

## 2 MATERIALS AND METHODS

### 2.1 Animal care

The transgenic zebrafish lines *Tg (cldnb: lynGFP)* and *Tg (brn3c:mGFP)* were bred and maintained in the zebrafish facility of Fudan University following standard procedures. The temperature of the constant temperature incubator was set to 28.5°C for proper growth of the spawning embryos in embryo medium (EM). All animal ethics were approved by the Institutional Animal Care and Use Committee of Fudan University, Shanghai. Hours post‐fertilization (hpf) was used to label the age of embryos and larvae after birth. The embryonic development stages were segmented according to the guideline of Kimmel et al.^7^ The embryos were anaesthetised in 0.02% MS‐222 (Sigma‐Aldrich) before operations.

### 2.2 Morpholino injections

For transcriptional silencing of Dnmt1, morpholinos were designed and produced from Gene Tools, Inc. and were diluted and stored in sterile water at a 1 mM final concentration for subsequent operations. The sequences were exhibited as follows: Dnmt1 (5′‐ACAATGAGGTCTTGGTAGGCATTTC‐3′) and standard control MO (Con‐MO) (5′‐CCTCTTACCTCA GTTACAATTTATA‐3′).

One‐cell stage of embryos was stretched in a series of straight lines, and then different doses of morpholino were injected into the embryos through a pulled pipet tip. After injection, the embryos were collected for subsequent experiments. To rescue the Dnmt1‐deficient embryos, Dnmt1 mRNA was synthesized and injected together with the morpholino.

### 2.3 Western blot analysis

Total protein of the whole embryos was extracted by RIPA lysis buffer (YOBIBIO, Shanghai, UBI1003). The protein concentrations were identified using A BCA protein kit from Thermo Fisher (Rockford, IL). Following separation on SDS‐polyacrylamide gels, proteins were transferred onto appropriate PVDF membranes (Immobilon‐P; Millipore, Bedford, MA, USA) following the manufacture's inductions. The membranes were blocked for 1 hour with 5% non‐fat dried milk in TBST at room temperature (RT), and then incubated overnight with Dnmt1 antibody (1:500 dilution, Abcam, UK, ab13537) or anti‐GAPDH antibody (1:1000 dilution, GNI, Japan, 4310‐GH) at 4 °C. The ECL kit (Pierce) was used to visualize the immunoreactive bands and the intensities of the bands were quantified with Fiji (National Institutes of Health).

### 2.4 WISH

WISH of zebrafish was performed according to the instruction of standard procedures.^25^ To be simplified, after fixation in 4% PFA, the collected embryos were washed in PBST and digested in 10 μg/mL proteinase K (storage in 20 mg/mL) for 24 hours–2 minutes, 30 hours–3 minutes, 36 hours–5 minutes and 48 hours–10 minutes. The samples were then prehybridized for over 4 hours at 65 °C in the prehybridization mix. Digoxigenin (DIG)‐labelled RNA probes were designed and prepared as described by the manufacturer of Roche (Mannheim, Germany). Primers for synthesizing the objective genes are listed in Table S1. After prehybridization, the solution was replaced with solute probes in the hybridization mix and reacted at 65 °C overnight. After incubation, the sections were washed in total four times in graded 2x SSCT at 65 °C every 10 minutes. After block in 2x BBR, the specimens were then incubated with alkaline phosphatase (AP)‐coupled anti‐DIG antibody at 4°C overnight. The embryos were rinsed and transferred to the 24‐well plate for staining with BM purple AP substrate (Roche, Mannheim, Germany) in the dark. Staining was stopped by two times of 10‐minutes wash in NTMT (Tris–HCl 1 M, NaCl 5 M, Tween‐20 and dd H_2_O). The specimens were photographed in 100% glycerol with a Nikon fluorescence stereomicroscope. All images were processed with Photoshop and Illustrator software.

### 2.5 BrdU staining and cell proliferation analysis

The immunocytochemistry staining of BrdU was used to mark the proliferative cells as described previously.^26^ To be brief, dechorionated embryos were incubated in 10 mM BrdU solution (Sigma–Aldrich, B5002‐5G) for 2 hours before collection. After washing in PBS to remove the BrdU, the embryos were fixed for 2 hours at RT in 4% PFA and rinsed in PBT‐2 (PBS containing 1% Triton X‐100). 2 N HCl was added to block non‐specific binding sites for 30 minutes at 37°C. After incubation with anti‐BrdU primary monoclonal antibody (1:200 dilution; Santa Cruz Biotechnology, Shanghai, sc‐32,323) overnight at 4°C, the samples were rinsed for several times and then added with the secondary antibody (1:200 dilution; Jackson ImmunoResearch Laboratories) for 2 hours co‐incubation at 37°C. After several times rinsing in PBT‐2, DAPI (1:800 dilution; Invitrogen) was added and incubated with the larvae for 20 minutes to recognize the nuclei. The images were photographed with a high‐definition confocal fluorescence microscope (TCS SP8, Leica) and processed with Photoshop and Illustrator software (2018, Adobe).

### 2.6 RNA‐seq analysis

Total RNAs were extracted from the experimental and control embryos at 48 hpf after deletion of yolk sac using RNeasy kit (Axygen) for RNA‐seq analysis. An Illumina HiSeq X Ten platform was used for library sequencing. Raw reads (fastq format) were firstly processed with FastQC and all the clean reads were available by filtering low‐quality, contamination and poly‐N reads from raw data, which were in high quality and were the base libraries of downstream analyses. Paired‐end clean reads were aligned to the reference genome using the Spliced Transcripts Alignment to a Reference (STAR) software. Differential expression analysis utilized the DESeq (2012) R package. *p*‐value <0.05 and >2, and FDR <0.01 indicated significant differential expression. KEGG pathway enrichment analyses of DEGs were performed with R on the basis of hypergeometric distribution. Functional and pathway enrichment analyses were performed based on KEGG pathway database.

### 2.7 WGBS analysis

Genomic DNAs (gDNAs) were extracted from the experimental and control embryos at 72 hpf after deletion of yolk sac with the AllPrep DNA/RNA Mini Kit (Qiagen). After quality validation, and addition of positive control DNA, the gDNAs (5 μg) were fragmented to 200–300 bp fragments with the Covaris S220 Focused‐ultrasonicator (Covaris). Fragment terminal repair, A‐ligation and methylation sequencing adapter ligation were then conducted using the TruSeq DNA Library Preparation Kit (Illumina). Unmethylated cytosines were converted to bisulphite using the EZ DNA Methylation Gold Kit (Zymo Research). Bisulfite‐treated DNA was subsequently PCR amplified to enrich both‐end adapter fragments. Purified libraries were quantified by the Agilent 2100 Bioanalyzer (Agilent Technologies). Paired‐end sequencing of pooled libraries was done using the HiSeq 6000 platform (Illumina). FastQC software was used to assess the quality of the raw data. After filtering the low‐quality data, the sequence was aligned to the reference zebrafish genome using Bismark software. The Q20, Q30, GC content of the clean data and the paired map rate and duplication rate were calculated. The violin diagrams were plotted for comparison in the overall methylation level between the two groups. The methylation level of C base is calculated according to the following formula: methylated cytosines/all cytosine sites in special regions *100. Differentially methylated region (DMR) analysis was conducted by R package.

### 2.8 Statistical analysis

GraphPad Prism (version, 8.0c, San Diego, CA, USA) was used for statistical analysis. One‐way ANOVA was used for comparisons between more than two groups, while an unpaired two‐tailed Student *t*‐test was performed for two‐group comparisons. Statistics are all presented as mean ± SEM (standard error of mean), and *p*‐value<0.05 was determined as significantly different.

## 3 RESULTS

### 3.1 Dnmt1 is expressed in the inner ear and neuromasts of zebrafish

**FIGURE 1:**
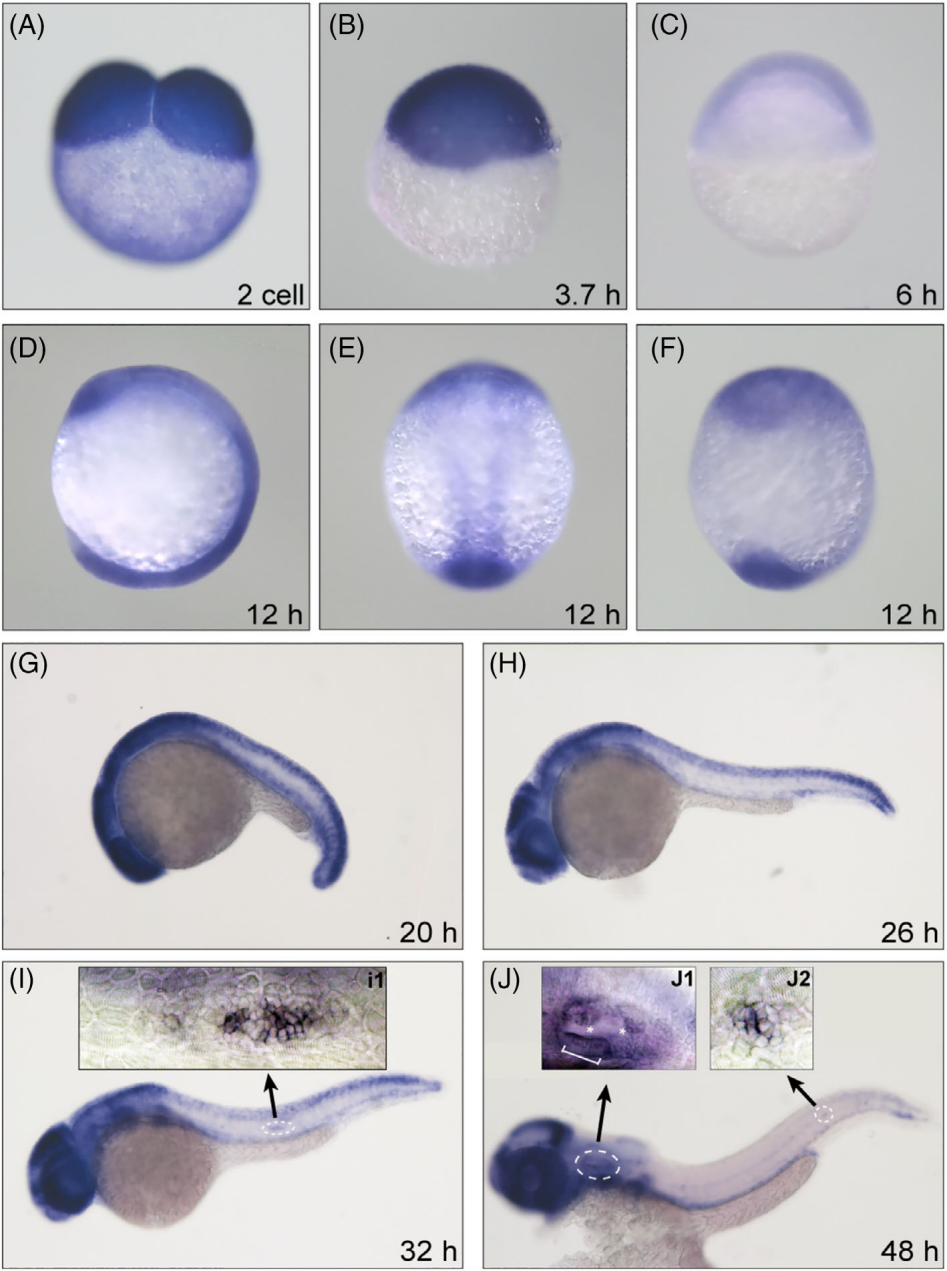
Dnmt1 is required for the development of zebrafish inner ear and pLL. The WISH staining delineates a persistent expression of Dnmt1 at two‐cell stage (A), oblong stage (B), shield stage (C), segmentation period (D: lateral view, E: dorsal view, F: ventral view at 12 hpf and G: lateral view at 20 hpf). The high expression levels of Dnmt1 are concentrated on the migrating primordium (H and I), inner ear in the head region and deposited neuromasts in the posterior lateral line (J) from 26 to 48 hpf. The magnification‐times images of primordium (I1), inner ear (J1) and neuromast (J2) structure indicate a strong expression of Dnmt1. The white bracket outlines sensory maculae HCs and white asterisks label semicircular canals (J1)

To ascertain whether Dnmt1 is required during zebrafish embryogenesis, we first assessed Dnmt1 expression pattern in zebrafish through WISH. The expression of Dnmt1 was detected as early as the two‐cell stage (Figure 1A), and maintained throughout the pivotal stages before segmentation including embryonic blastula (Figure 1B) and shield stage (Figure 1C). At the 6‐somite stage around 12 hpf, strong staining of Dnmt1 was concentrated in the upper optic region and lower Kupffer's vesicle region from three different views (Figure 1D: lateral view; 1E: dorsal view; and 1F: ventral view). Dnmt1 expression was mainly sustained in the encephalic optic and otic region and the caudal region at 20–26 hpf (Figure 1G‐H). We observed the expression of Dnmt1 in the migrating primordium from 26 to 32 hpf (Figure 1H‐I). Dnmt1 was also expressed in the deposited neuromasts at the dorsal posterior lateral line at 48 hpf (Figure 1J). The enlarged details of *dnmt1* expression pattern in the inner ear and lateral line were delineated in the small boxes (Figure 1I1, 1 J1 and 1 J2). Altogether, we traced the developmental process of zebrafish using WISH and described an on‐going and high expression of Dnmt1 in the otic vesicle and posterior lateral line. Our results suggest that Dnmt1 is involved in the development of auditory organs of zebrafish.

### 3.2 Dnmt1 is requisite for proper deposition of lateral line neuromasts

**FIGURE 2:**
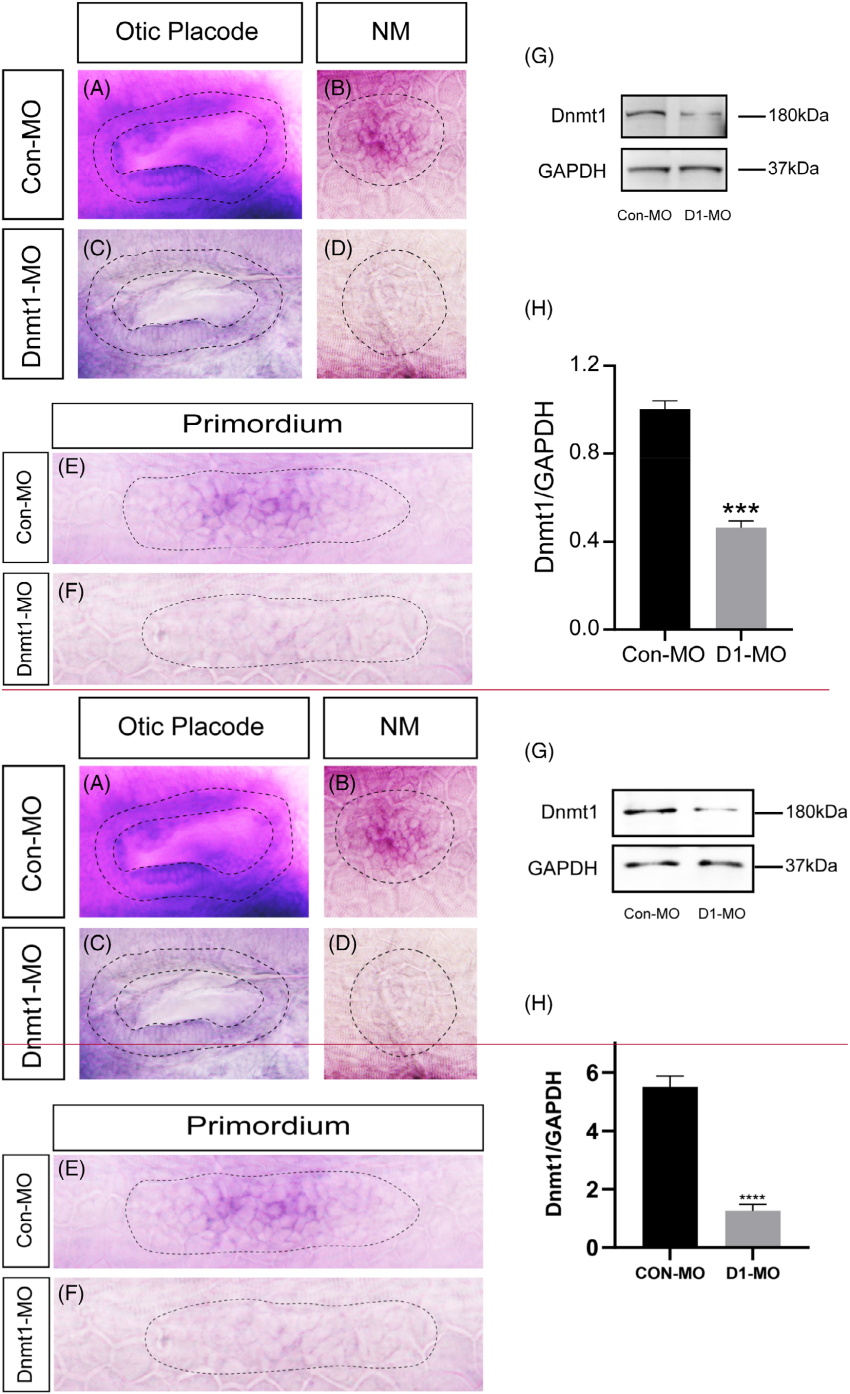
The expression of dnmt1 is markedly downregulated after knocking down of Dnmt1. A‐F, In situ staining of dnmt1 is uniformly downregulated in Dnmt1‐MO embryos compared to the controls by WISH at different stages. The black dotted lines outline the otic vesicle (A, C) and neuromast (B, D) at 48 hpf respectively. The black dotted lines in E‐F outline the primordium at 32 hpf. G‐H, The protein blotting of Dnmt1 is significantly decreased by Dnmt1 morpholino injection both in the band intensity (G) and the quantification analysis (H)

To investigate the function of Dnmt1 during the zebrafish hearing organ morphogenesis, Dnmt1‐MO was injected to block the gene expression of Dnmt1. The ISH analysis demonstrated a severely reduced expression of Dnmt1 in otic placode, neuromasts and primordium of zebrafish in Dnmt1‐MO group compared to the Con‐MO group (Figure 2A‐F). Moreover, the western blotting tests confirmed a successful reduction of *dnmt1* in the Dnmt1 morpholino knockdown siblings in comparison with the Con‐MO‐injected embryos (Figure 2G‐H), indicating a potent efficacy of Dnmt1‐MO in downregulating the transcript level of Dnmt1.

**FIGURE 3:**
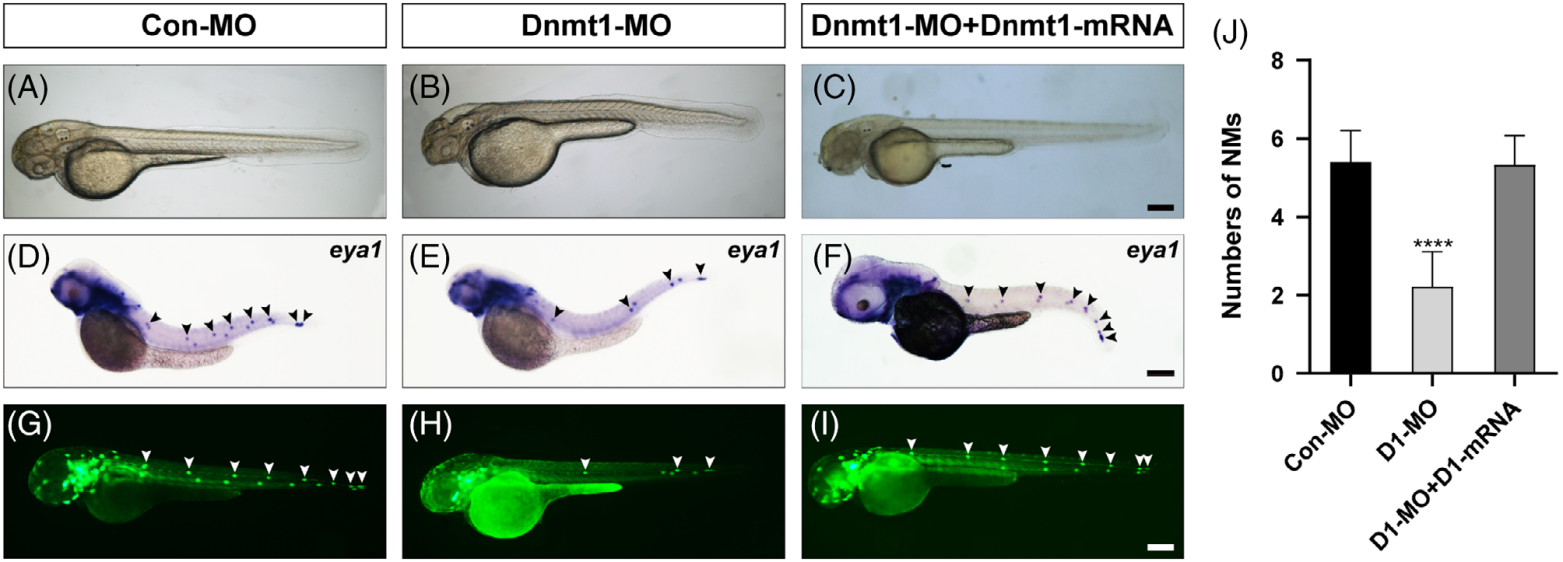
Dnmt1 is required for neuromast deposition in zebrafish posterior lateral line. A‐B, At 48 hpf, the gross morphology of wild‐type embryos and Dnmt1‐deficient embryos. D‐E, Reduced number of neuromasts and extended distance between neuromasts are found in the staining of *eya1* in lateral line neuromasts when compared the controls (D) with the Dnmt1 morphants (E). G‐H, The transgenic zebrafish line *cldnb:lynGFP* confirms the results observed in WISH. (C, F, I) Successful rescue of morphology, *eya1* staining and green fluorescence labelled neuromasts are achieved in mRNA and MO co‐injection groups. The black arrowheads in D‐F, and the white arrowheads in G‐I both mark the PLL neuromasts. Scale bars mark the 200 μm scale. (J) Statistic analysis of the number of posterior LL neuromasts at 48 hpf in controls (n = 117), Dnmt1‐deficient embryos (n = 159) and Dnmt1‐MO + mRNA members (n = 159). ****p < 0.0001

We first observed the gross morphology of zebrafish, and we did not detect obvious phenotype malformation as a whole after injection with Dnmt1 antisense morpholino using the white light field microscope (Figure 3A‐B). Next, we examined the expression of a neuromast marker, the eyes absent‐1 gene (*eya1*),^27^ to visualize the phenotypic changes after downregulating Dnmt1. As shown in Figure 3D‐F, *eya1*‐labelled neuromasts were distinguished along the trunk and tail of zebrafish in different groups. The gross appearance showed a significant reduction in the number of pLL neuromasts in the Dnmt1 morphants compared to the controls (Figure 3D‐E). We confirmed this finding using the *cldnb: lynGFP* zebrafish, in which the neuromasts are labelled with green fluorescence, and deficiency of the first neuromast was also detected after knocking down of Dnmt (Figure 3G‐H). Besides, the disappeared neuromasts resulted in extended separation distance between neuromasts in Dnmt1‐deficient embryos compared to that of Con‐MO injection (Figure 3D‐E and 3G‐H). The quantitative analysis showed that there were 5.4 ± 0.074 (n = 117) neuromasts in the trunk region and two or three caudal neuromasts in the controls at 48 hpf, while the number of pLL neuromasts along the body but not the terminal neuromasts decreased significantly to 2.2 ± 0.071 (n = 159) in Dnmt1‐MO‐injected embryos (Figure 3J). To further verify the role of Dnmt1 during zebrafish lateral line development, we injected mRNA of Dnmt1 together with the specific antisense morpholino and observed a total rescue in the number of neuromasts without inducing any deformity in contrast to the controls and Dnmt1‐MO morphants (Figure 3C, F, I and J). Our results suggest that Dnmt1 is required for proper neuromasts deposition of the lateral line.

### 3.3 Dnmt1 inhibition evidently suppresses PLL cell proliferation behaviour

**FIGURE 4:**
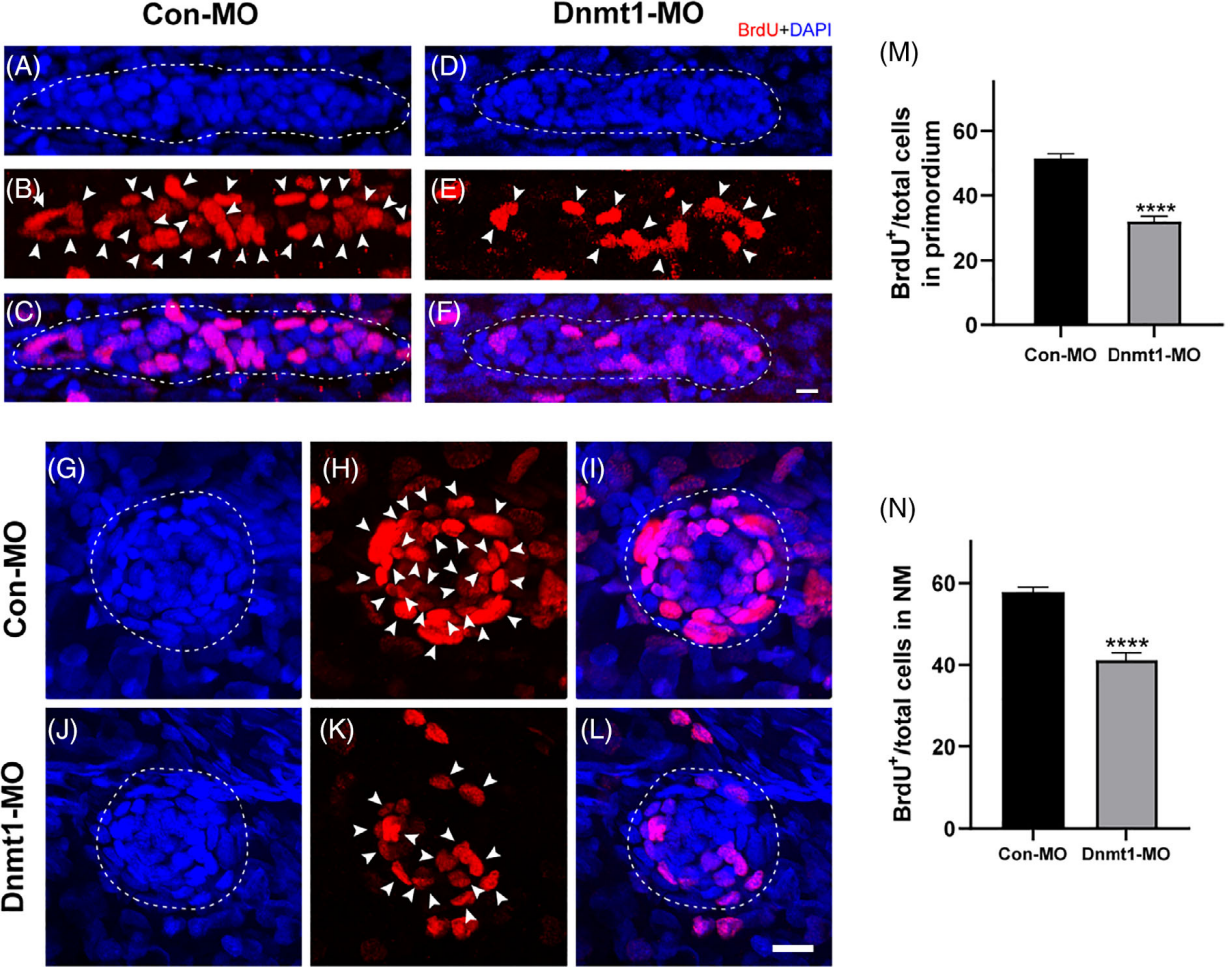
Downregulated Dnmt1 represses cell proliferation during primordium migration and neuromast formation. A‐L, Representative images show the comparison in number of BrdU‐labelled proliferating cells between controls embryos and Dnmt1‐deficient mutants at 32 hpf in primordium (A‐F) and at 48 hpf (G‐L) in neuromasts. Scale bars mark the 10 μm scale. M‐N, Significant differences in quantification of BrdU index in control embryos (n = 32) and Dnmt1‐MO embryos (n = 31) were labelled. The arrowheads in B, E, H, K mark the proliferative cells. Dotted lines in A, C, D, F outline the primordium, and dotted lines in G, J, I, L outline the neuromast. Data are recorded as mean ± SEM. ****p < 0.0001

Cell proliferation and migration are the fundamental events during the early stage of embryogenesis in zebrafish.^28^ In the Dnmt1‐deficient embryos, we detected less BrdU‐positive cells compared to that of the controls at 32 hpf, a time point when the first neuromast has deposited and the primordium is migrating (Figure 4A‐F). The BrdU index defined as the ratio of BrdU‐labelled cells to the total number of cells is used to assess the proliferative capacity of cells. As shown in Figure 4M, the BrdU index of primordium was significantly decreased in Dnmt1‐deficient embryos in comparison with the controls. Similarly, severe reduction in the number of BrdU‐positive cells was also examined in the Dnmt1‐MO PLL neuromasts compared to the Con‐MO larvae at 48 hpf when the migration of collective cells completed (Figure 4G‐L). Equally, the BrdU index in Dnmt1‐MO neuromasts was remarkably reduced in contrast to that in the Con‐MO larvae (Figure 4N). Our results suggest that the cell proliferation process is significantly inhibited after knocking down Dnmt1 both in the migrating primordium and deposited neuromasts of zebrafish PLL.

### 3.4 DNMT1 controls the PLL development by regulating Fgf, Wnt and chemokine signalling pathways

**FIGURE 5:**
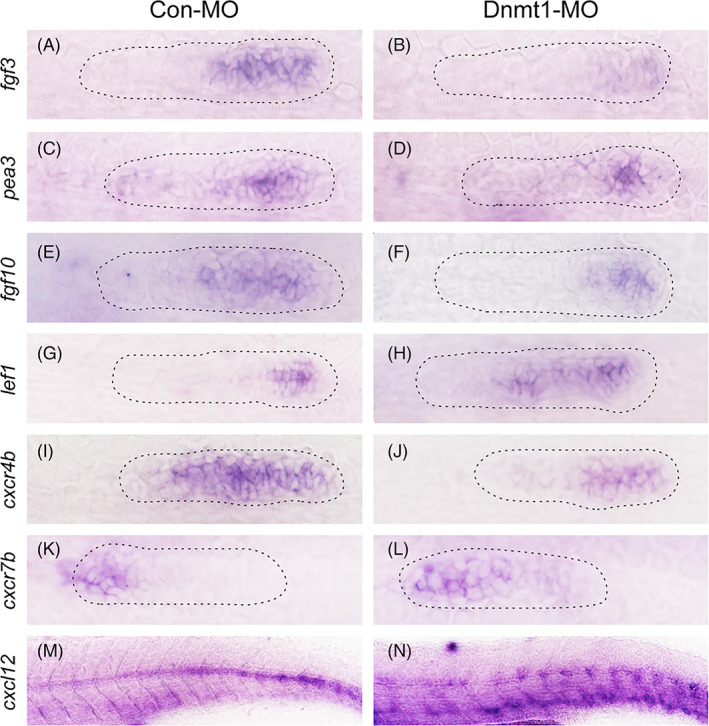
Dnmt1 depletion represses Fgf signalling and the chemokine superfamily in zebrafish primordium. Corresponding decreased expression of Fgf signalling components including *fgf3*, *pea3* and *fgf10* are presented in Dnmt1 morphants compared to the control siblings at 32 hpf (A‐F). On the contrary, the expression of *lef1*, a target gene of Wnt signalling, is increased in Dnmt1‐deficient embryos (G‐H). The expression levels of chemokine ligands are discrepant that *cxcr4b* transcript level is decreased (I‐J) but *cxcr7b* transcript level is increased (K‐L) in Dnmt1‐MO morphants in comparison with Con‐MO samples. The staining of *cxcl12* is completely discontinuous along the intermediate line of the trunk in Dnmt1‐MO group compared to the controls (M‐N). The primordium appearance is shaped by dotted lines. Each group has eight zebrafish, and the results are repeated for three times

Recent works have shed light onto the crucial roles of signalling pathways in regulating primordium formation and migration, in particular Fgf and Wnt/β‐catenin signalling and their cross talks.^4^, ^28^, ^29^, ^30^ To find out the behind mechanisms of Dnmt1 in modulating development of the lateral line organ in zebrafish, we proposed that whether Dnmt1 worked through Fgf and Wnt signalling pathways. We detected the expression of Fgf family members including *fgf3*, *pea3* and *fgf10* in controls at 32 hpf; however, their expression levels were all severely downregulated after Dnmt1 deficiency (Figure 5A‐F). Conversely, we found an extended expression of *lef1*, a Wnt/β‐catenin target gene, in the deficient model of Dnmt1 compared to controls, indicating an inverse regulation in comparison with that of Fgf signalling in primordium migration (Figure 5G‐H).

CXCL12 (SDF‐1α) is a chemokine family member that interacts with CXCR4 and CXCR7 on the surface of cell, playing pivotal roles in the progression of inflammation, cell directional migration and proliferation.^28^, ^31^ We next examined whether the *cxcl12‐cxcr4b/cxcr7b* axis was also affected when Dnmt1 is deficient. At 32 hpf, *cxcr4b* was detected to be broadly expressed in a major part of the primordium, while *cxcr7b* was narrowly expressed in the trailing zone in the control larvae. In Dnmt1‐MO morphants, the expression level of *cxcr4b* was significantly downregulated, whereas the expression of *cxcr7b* was upregulated compared to the controls (Figure 5I‐L). Normally, *cxcl12* was expressed in a narrow and continuous stripe pattern along the horizontal myoseptum of zebrafish body, however the expression of *cxcl12* was totally disrupted in Dnmt1 morphants showing a discontinuous stripe‐like pattern (Figure 5M‐N).

Altogether, the ISH data indicated that Dnmt1 was required for the early development of zebrafish by regulating Fgf and Wnt signalling pathways together with the chemokine ligands and receptors.

### 3.5 Dnmt1 knockdown in zebrafish induces severe otic deformities

**FIGURE 6:**
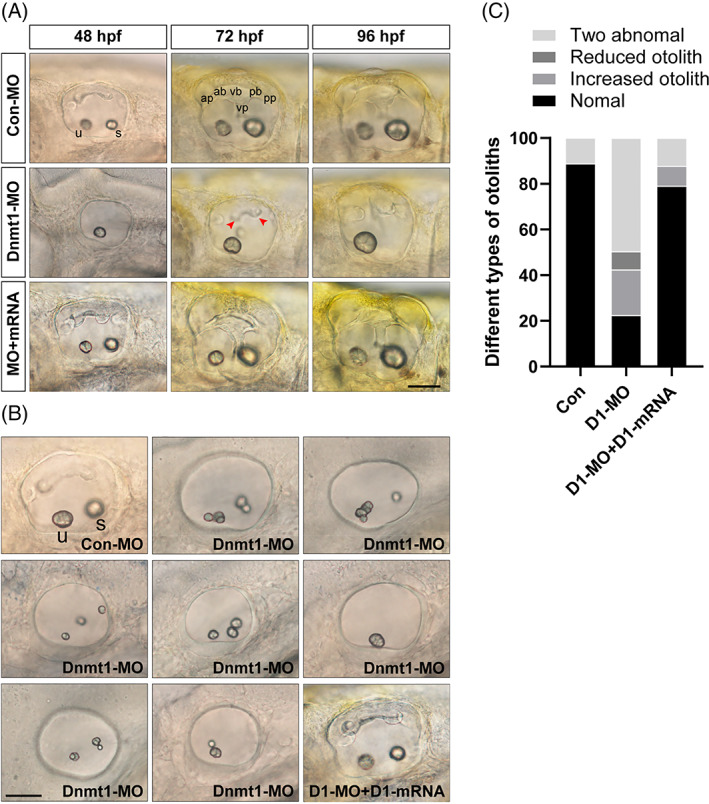
Knockdown of Dnmt1 disrupts the normal development of semicircular canals and otolith organs. A, Knockdown of Dnmt1 fails to form normal semicircular canals as well as otolith organs from 48 hpf to 96 hpf. Anterior, posterior and lateral (ventral) semicircular canals together with two otolith organs are well‐structured in controls and in the mRNA rescue larvae, while in Dnmt1‐MO morphants the normal semicircular canals are absent and the malformation of otolith maintain from 48 to 96 hpf. B, The inner ear structure of zebrafish at 48 hpf in white light in controls shows two normal otolith organs labelled as the anterior utricle and the posterior saccule, and upsides epithelial thickening. Multiple abnormal otolith phenotypes after Dnmt1 depletion are found at 48 hpf: increased number malformation, decreased number malformation and two abnormal otoliths. Successful rescue of phenotypic abnormalities is found in Dnmt1‐MO + Dnmt1 mRNA groups. C, Different types of deformities and their percentages are compared among the three groups (n = 62, 420 and 81 in controls, Dnmt1‐MO morphants and rescue embryos respectively). u: utricle, s: saccule, ap: anterior protrusion, ab: anterior bulge, vb: ventral bulge, vp: ventral protrusion, pb: posterior bulge and pp: posterior protrusion. Red arrow heads show the abnormal fusion of anterior and posterior protrusions. All images are lateral views with the anterior to the left and the dorsal side up. Scale bars are 50 μm (A, B)

To further study whether Dnmt1 deficiency affect the later development of semicircular canals and otolith organs, we took photos of the inner ear in succession from 48 hpf to 96 hpf. At 48 hpf, the normal cristae and epithelial thickening were completely depleted when Dnmt1 is deficient (Figure 6A). At 72 hpf and 96 hpf, the projections from the epithelium failed to fuse into pillar‐shaped canals and only short and unshaped protuberances were recognizable in Dnmt1‐MO morphants (Figure 6A). Furthermore, we detected successful rescue of semicircular canals deformation in the Dnmt1 mRNA and morpholino co‐injected experimental embryos (Figure 6A).

In addition, otolith organ malformations in quantity, size and location were also detected in the Dnmt1 morphants at 48 hpf (Figure 6B). We divided the deformed otoliths into three types according to the number aberrance, and as shown in Figure 6B and C, 49.8% embryos showed two abnormal otoliths, 7.8% embryos showed one solo otolith, 20.0% embryos showed three or more otoliths, while only 22.4% embryos showed normal appearance in Dnmt1‐MO groups (n = 420). In contrast, over 85% embryos of the control group (n = 62) exhibited normal otoliths. To verify the Dnmt1 activity in zebrafish inner ear, we co‐injected mRNA of Dnmt1 with the morpholino solution and detected normal otolith phenotype as shown in Figure 6B. Surprisingly, the rescue efficiency of inner ear malformation in Dnmt1‐MO + Dnmt1 mRNA‐co‐injected embryos (n = 81) was very high, that the rate of normal otoliths raised to 79.0%, while the ratio of two abnormal deformities decreased to 12.3% (Figure 6C).

**FIGURE 7:**
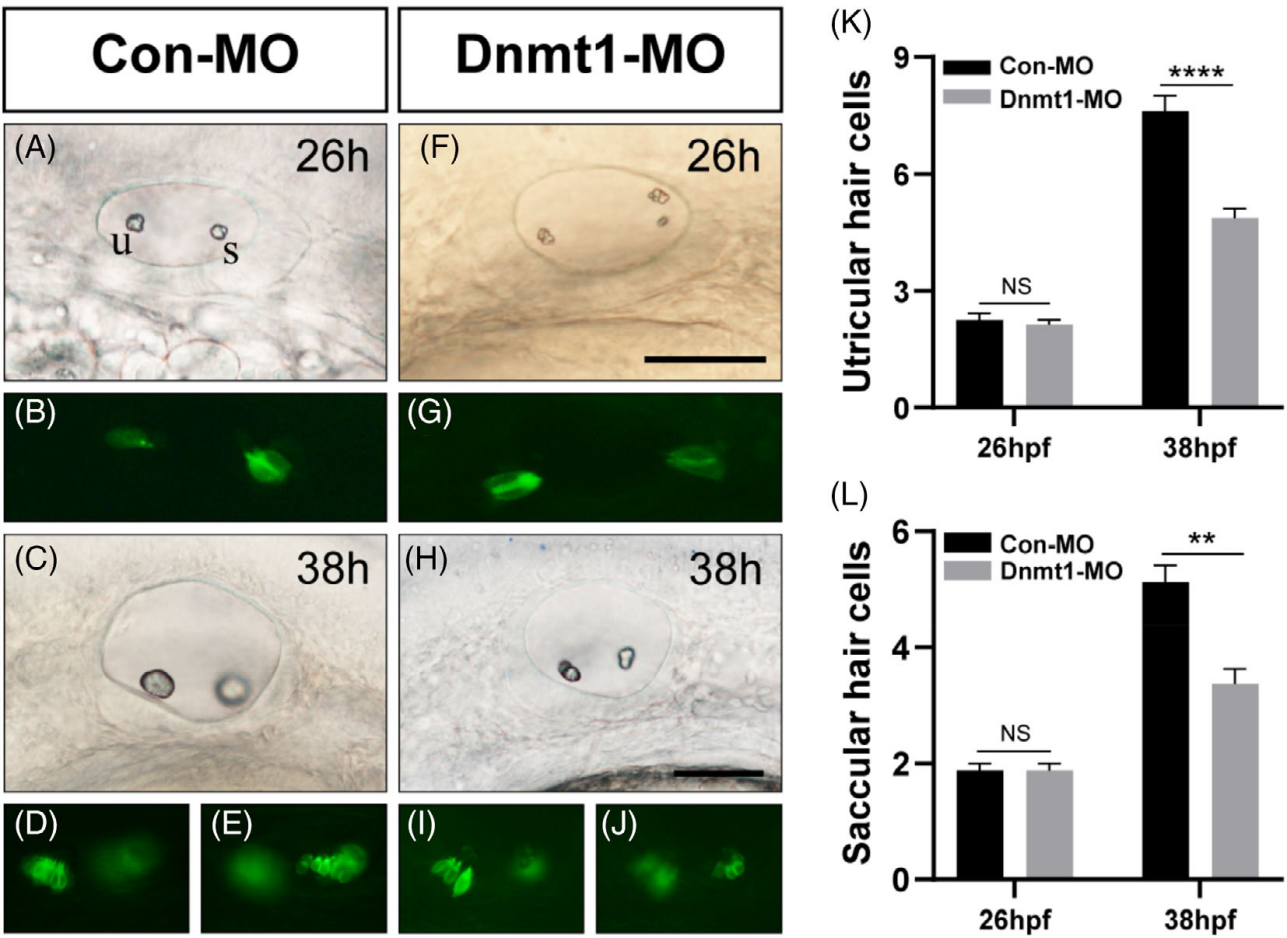
The HC differentiation both in utricle and saccule are interfered by Dnmt1 inhibition. At 26 hpf, the number of progenitor HCs are comparable in Con‐MO and Dnmt1‐MO group (A‐B, F‐G, K). At 38 hpf, reduced numbers of HCs are observed in Dnmt1‐MO defects compared to the controls (C‐D, H‐J, L). Scale bars are 50 μm (A, C, F and H). Data are recorded as mean ± SEM. **p < 0.01, ****p < 0.0001

To determine whether the inner ear HC differentiation is interfered by Dnmt1 inhibition, we chose *Tg (brn3c:mGFP)* transgenic zebrafish to visualize HCs in GFP.^32^ However, we failed to find any difference in the number of progenitor HCs at 26 hpf between the controls and Dnmt1‐MO morphants both in the utricle and saccule (Figure 7A‐B, F‐G, K; Con‐MO: 2.3 ± 0.16 utricular HCs and 1.9 ± 0.13 saccular HCs, n = 8 and Dnmt1‐MO: 2.1 ± 0.13 utricular HCs and 1.9 ± 0.13 saccular HCs, n = 8 respectively). We then elongated the observation window and detected remarkably decreased number of utricular HCs together with saccular HCs in Dnmt1‐MO defects compared to the control siblings at 38 hpf, a time when the progenitor HCs differentiated into mature HCs in the two poles of zebrafish inner ear (Figure 7C‐E, H‐J, L; Con‐MO: 7.63 ± 0.38 utricular HCs and 5.13 ± 0.30 saccular HCs, n = 8 and Dnmt1‐MO: 4.88 ± 0.23 utricular HCs and 3.38 ± 0.26 saccular HCs, n = 8 respectively). Thus, inhibition of Dnmt1 induced malformed inner ear‐genesis including abnormal otoliths and the failure to form semi canals, indicating a pivotal role of DNA methylation in the development of auditory and vestibular organs.

### 3.6 Dnmt1 is involved in zebrafish inner ear embryogenesis through regulation of pax family and Fgf signalling

**FIGURE 8:**
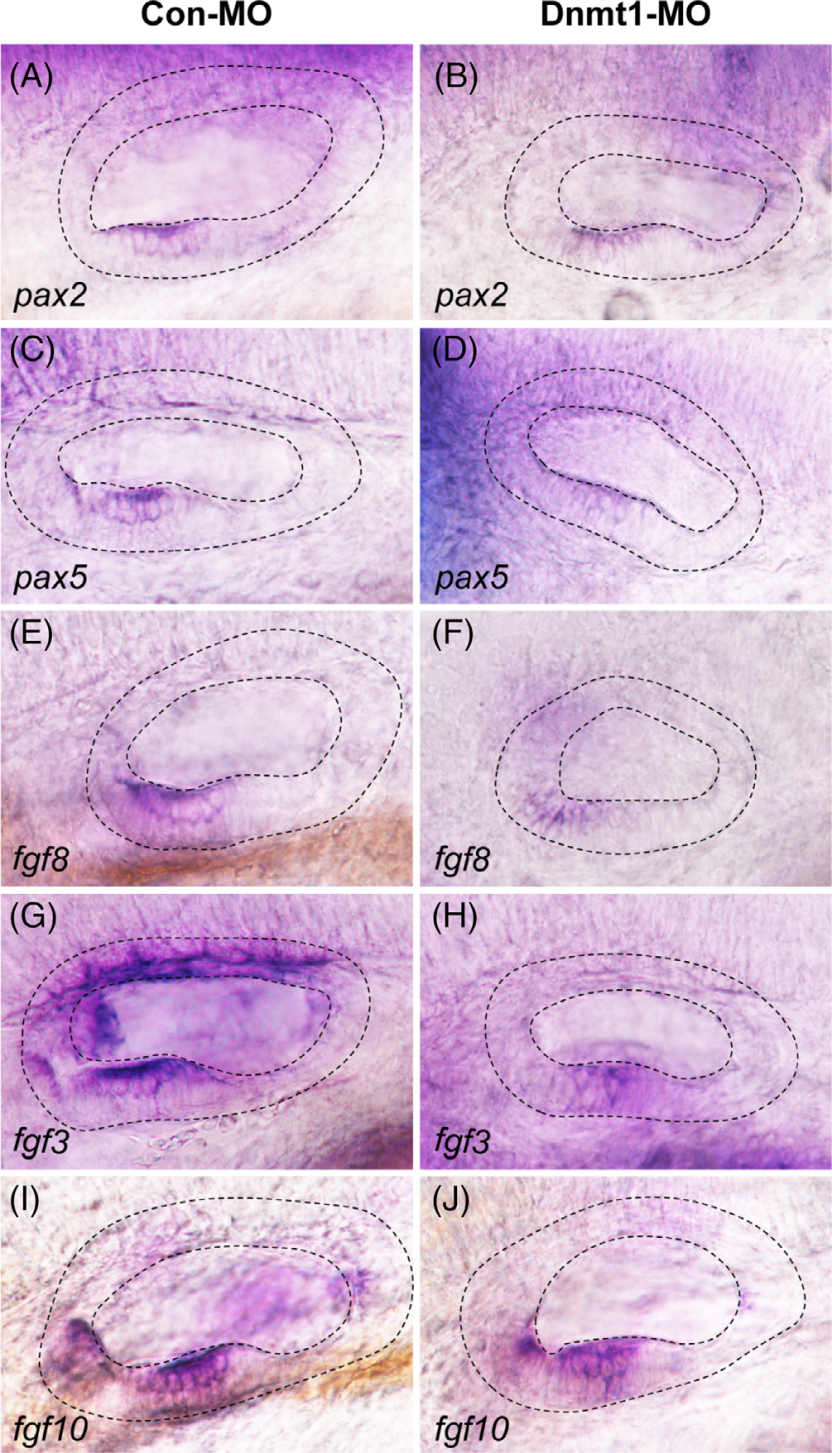
Changes in the expression of otic placode marker genes after Dnmt1 knockdown. In situ staining of *pax2* and *pax5* are all downregulated in Dnmt1‐MO embryos compared to the controls by WISH at 48 hpf. The black dotted lines outline the otic vesicle. The Fgf signalling ligands *fgf8*, *fgf3* and *fgf10* are in lower expression in Dnmt1‐MO embryos compared to the controls at 48 hpf

To investigate the underlying mechanisms of Dnmt1 in regulation of the development of inner ear, we examined the expression level of ear‐specific marker genes, including *pax2* and *pax5* ^6^, after Dnmt1 knockdown. *Pax2* was expressed anterior‐ventrally in the OV at 48 hpf in control embryos, whereas the expression of *pax2* significantly reduced after knockdown of Dnmt1 (Figure 8A‐B). Besides, the dotted line‐labelled otic domain also decreased in the Dnmt1 morphants compared to controls (Figure 8A‐B). Similarly, we found a diminished expression of *pax5* in the ventral‐medial region of OV in Dnmt1‐deficient embryos compared to that of controls at 48 hpf (Figure 8C‐D).

The Fgf signalling is involved in the induction of otic organ.^6^ In our previous study, we have detected the expression of *fgf3*, *fgf8* and *fgf10* genes in zebrafish OV.^33^ We next examined the expression of Fgf family components after knocking down Dnmt1. As shown in Figure 8, *fgf8*, *fgf3* and *fgf10* staining were detected in the anterior‐ventral maculae of OV at 48 hpf in the control embryos. On the contrary, the expression levels of all the three members were consistently decreased in situ in Dnmt1‐MO embryos (Figure 8E‐J).

### 3.7 Dnmt1 regulates the transcript levels of *cdkn1a* and t*p53* during zebrafish development

**FIGURE 9:**
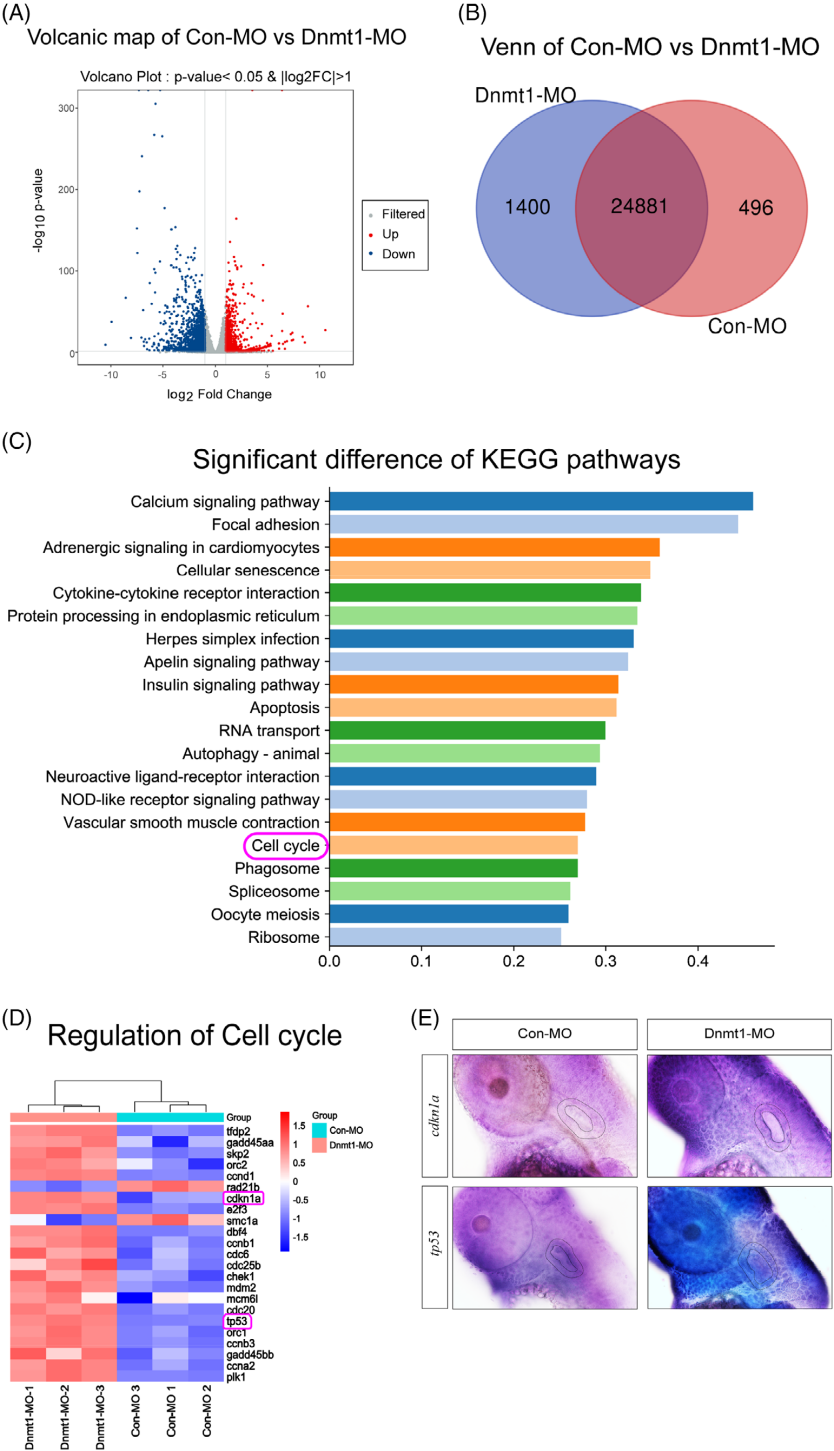
RNA‐Seq analysis uncovers cell cycle genes as the key regulator of Dnmt1 inhibition during otic development of zebrafish. A, Volcanic plot analysis displays all the DEGs in the two groups. B, Venn map further labels the unique and overlapping number of DEGs in Con‐MO and Dnmt1‐MO groups. C, KEGG enrichment analysis exhibits the top 20 signalling pathways statistically different between Dnmt1 knockdown samples and the controls. D, Cluster analysis of DEGs on cell cycle pathway between the two groups. E, The ISH data confirm both *cdkn1a* (*p21*) and *tp53* upregulated after knockdown of Dnmt1 compared to controls at 48 hpf

To reveal the downstream targets of Dnmt1 in regulating the early stage of development of zebrafish, we next performed differential gene expression analysis through RNA‐seq. Volcanic plot analysis displayed all the differentially expressed genes (DEGs) including the upregulated genes (red) and downregulated genes (blue) in Dnmt1‐deficient larvae compared to the controls (Figure 9A). To know the number of DEGs between the Dnmt1‐MO group and Con‐MO group, Venn map was plotted that 1400 unique DEGs were found in Dnmt1‐MO larvae, 496 unique DEGs were distinguished in Con‐MO group and 24,881 common difference genes expressed in the two groups were labelled in the middle overlapping ellipse region (Figure 9B). KEGG pathway analysis demonstrated the top 20 signalling pathways which were significantly different between Dnmt1 knockdown samples and the controls (Figure 9C). Combining our finding that cell proliferation was significantly inhibited after knocking down of Dnmt1, we focused on changes in cell cycle genes from the top 20 differently expressed pathways. The cluster analysis of DEGs in cell cycle pathway indicated that *cdkn1a* (*p21*) and *tp53* were markedly highly expressed in Dnmt1‐MO morphants compared to those in Con‐MOs (Figure 9D). The ISH analysis confirmed the result by RNA‐seq that *cdkn1a* (*p21*) and *tp53* were both upregulated in OV (outlined by dotted lines) after knockdown of Dnmt1 compared to controls at 48 hpf (Figure 9E).

**FIGURE 10:**
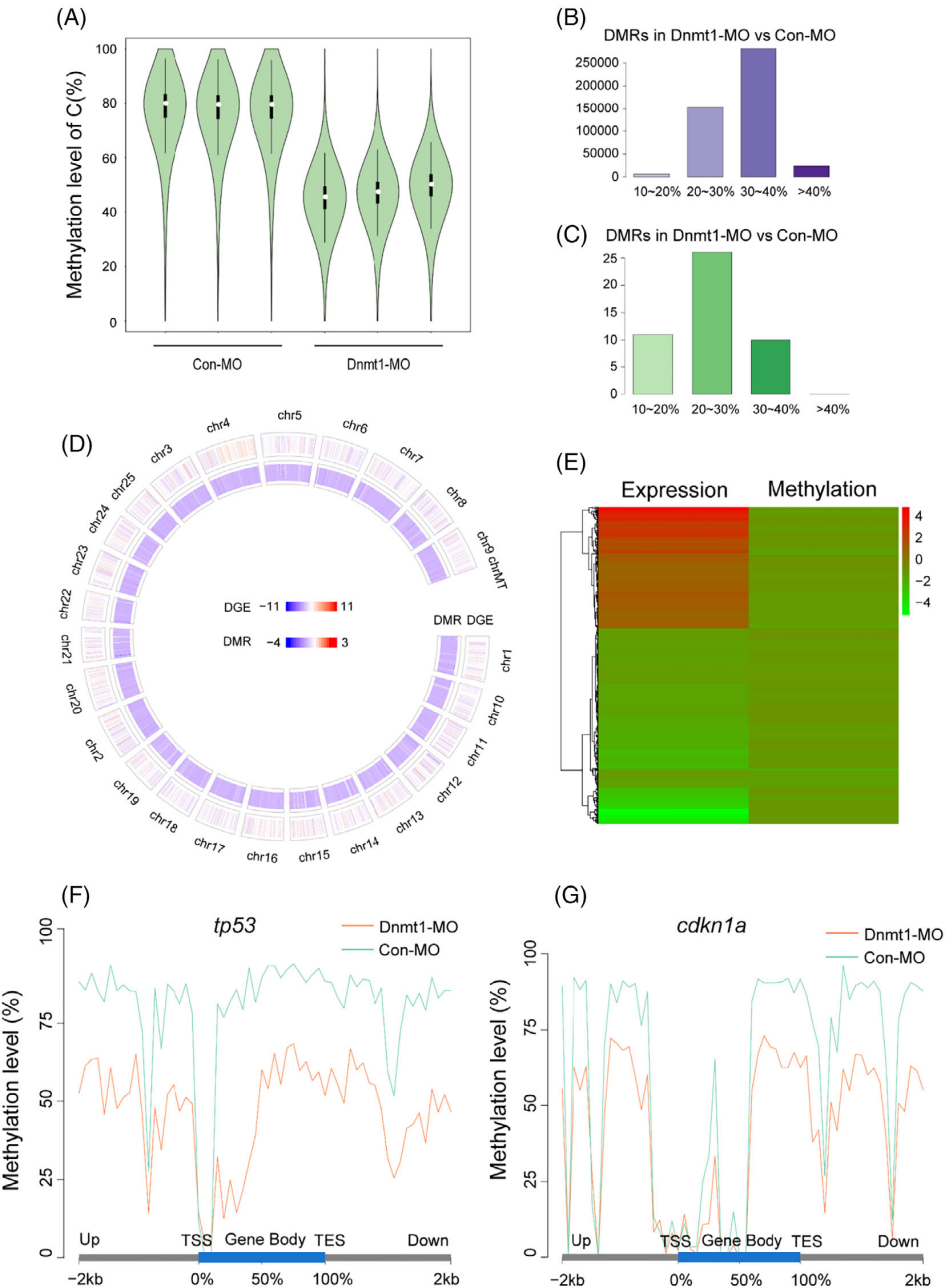
WGBS analysis detects global hypomethylation after knocking down Dnmt1. A, The violin diagrams show global methylation levels in Con‐MO group and Dnmt1‐MO group with three repeats. The white dot indicates median, the black solid frame indicates interquartile range (IQR), the thin black line indicates data range and the width of violin indicates the distribution density. B‐C, The number of DMRs with hypomethylation (B) and hypermethylation (C) in Dnmt1‐MO group compared to Con‐MO group. The abscissa represents different methylation levels. D, Circos figure displays the general distribution of DEGs and DMRs in genome Dnmt1‐MO group compared to Con‐MO group. The chromosome number, the DEGs (RNA‐Seq) and DMRs (CpG) are present from the outer ring to the inner ring. Red indicates upregulation, blue indicates downregulation; the darker the colour, the greater the difference. E, The heat map shows the common DEG‐ and DMR‐related genes in Dnmt1‐MO group compared to Con‐MO group. Red indicates upregulation and green indicates downregulation. F‐G, The methylation levels of *tp53* and *cdkn1a* in the functional region are all significantly reduced in Dnmt1‐MO morphants compared to the Con‐MOs. TES, transcription end site; TSS, transcription start site

To further investigate the causal relationship between Dnmt1 silence and the alterations in *cdkn1a* and *tp53*, we performed WGBS analysis to examine the global methylation status and the methylation levels of *tp53* and *cdkn1a* in the functional region. As shown in Figure 10, the total methylation levels of C base were all significantly reduced in three repeats after knocking down of Dnmt1 compared to the Con‐MO group (Figure 10A), indicating a powerful efficacy of Dnmt1‐MO in decreasing the methylation level. Moreover, thousands of DMRs with hypomethylation and several DMRs with hypermethylation were also found in Dnmt1‐MO morphants compared to the Con‐MO group (Figure 10B‐C). The general methylation level from DEG and DMR analysis in Dnmt1‐MO group was further confirmed to be lower than that in the Con‐MO group (Figure 10 D‐E). We further found significant reduction in the methylation levels in the functional region of both *tp53* and *cdkn1a* (Figure 10F‐G). Since the hypomethylation level in the upstream and promoter region indicates high transcript level, the WGBS analysis on *tp53* and *cdkn1a* suggested a primary effect of Dnmt1 inhibition.

## 4 DISCUSSION

Our previous study explored that using Dnmt1 inhibitor RG108, or specific siRNA target Dnmt1, could provide protective function against noise‐induced hearing loss in C57BL/6J mice, indicating a potential therapeutic effect of DNA hypomethylation on hearing impairment.^34^ However, the role of Dnmt1 in the development of auditory organ is unclear. In this study, we demonstrated that Dnmt1 was required for the development of zebrafish through a loss‐of‐function strategy using special antisense morpholino targeting Dnmt1. The number of PLL neuromasts was remarkably decreased and the first neuromast often failed to deposit in Dnmt1‐MO embryos. Cell proliferation was severely inhibited after Dnmt1 deficiency in the primordium and neuromasts compared to that of Con‐MO embryos. We also found abnormal otoliths and reduced utricular and saccular HC differentiation in the OV of Dnmt1‐MO morphants. Combining multiple methods including RNA‐seq, WGBS and ISH analysis, we confirmed that cell cycle genes, Fgf and Wnt signalling pathway, together with the chemokine family were differentially regulated by Dnmt1. To our knowledge, we first explore the function of Dnmt1 in the development of zebrafish auditory organs.

The reversible gene‐specific methylating alteration acts as an ideal therapeutic strategy in multiple diseases model, including cancer, thyroid disorders, autoimmune diseases and systemic inflammation.^35^ Altered methylation of specific genes has been widely involved in certain kinds of biological processes, especially in tumorigenesis and ageing.^19^, ^36^ Recent studies have linked the modifier of DNA methylation tightly with embryogenesis and development of inner ear. The inner ear sensory epithelium methylomes analysis underpins a low methylated region (LMR) as the regulator of GJB6, a critical gene of deafness. Besides, in the different stage of embryonic development, the DNA methylation level is dynamic over time and interacting with some signalling pathways such as Wnt and Notch.^37^ Demethylation of DNA by DNMT inhibitor induces mouse utricle stem cells to differentiate into sensory HCs, which are labelled by FM1‐43, a mechanosensory marker, indicating a requirement of epigenetic modification in cell fate determination of inner ear.^38^ DNMT3A and DNMT1 methyltransferases have been reported to be involved in age‐related hearing loss (ARHL) and noise‐induced hearing loss (NIHL).^39^

The primordium migration is a fundamental process during zebrafish embryonic development. In recent years, several mechanisms have been revealed to be corelated with the oriented migration of primordium.^28^, ^30^ As previously reported, blocking Fgf signalling by constructing *fgf3*; *fgf10* double morphants, Fgf special inhibitor SU5402 or heat‐shock inducible Fgfr1 depletion can severely reduce the migrating speed of primordium^30^ and change the directionality of migrating cells.^40^ Wnt/β‐catenin and Fgf signalling are also involved in primordium migration of zebrafish through regulating the chemokine signalling pathway components *cxcr4b* and *cxcr7b*.^28^ Consistent with the literature, this study revealed that knockdown of Dnmt1 inhibited the primordium migration and neuromast maturation through differently regulating the Fgf, Wnt and chemokine signalling pathways that the transcript levels of *fgf3, fgf10, pea3, cxcr4b* and *cxcl12* were significantly decreased, while the expression levels of *lef1* and *cxcr7b* were significantly increased in Dnmt1‐MO morphants compared to the controls. Similar with the findings in primordium, the expression levels of Fgf components *fgf3, fgf8* and *fgf10* and otic marker genes *pax2* and *pax5* were all reduced in OV of Dnmt1‐MO morphants. We further conducted WGBS analysis of these involved genes, and found that the methylation levels in functional region of *lef1, fgf3, fgf8, fgf10, pax2*, *pax5*, *cxcr4b* and *cxcr7b* were all significantly decreased in Dnmt1‐MO morphants compared to those in the controls (Figure S1 A‐H). Altogether, the results indicated that the upregulation of *lef1* and *cxcr7b* might be the primary effect of Dnmt1 deficiency, while the downregulation of other genes including *fgf3, fgf8, fgf10, pax2, pax5* and *cxcr4b* might be the secondary effects of Dnmt1 silence.

Our study uncovered that the negative cell cycle gene *cdkn1a* and tumour suppressor gene *tp53* were both significantly upregulated in Dnmt1‐deficient samples compared to the Con‐MO siblings. Further ISH experiment confirmed this result, that *cdkn1a* and *tp53* were highly expressed in the OV of Dnmt1‐depleted animals. This finding broadly supports the previous work that the proliferation of retinal stem cell (RSC) is severely repressed and cell death is elevated after *dnmt1* depletion.^41^ However, this study demonstrated that knockdown of Dnmt1 increased the expression level of *tp53*, while the study of Angileri et al.^41^ found that cell cycle inhibition was not in a *tp53*‐dependent manner.

In conclusion, our findings suggest the requirement of Dnmt1 for the normal formation of inner ear and lateral line system of zebrafish, and in vivo experiment using Dnmt1 knockout mouse model in the future might provide more clues of the potential function of Dnmt1 in the development of auditory system.

## CONFLICT OF INTEREST

There are no competing interests.

## AUTHORS' CONTRIBUTIONS

DMT, SMZ and ZWZ conceived the experiments, performed the majority of experiments, analysed the data and drafted the paper. CL, JNZ, RCY, CW, NZ, LJW and HFX performed partial experiments and acquired the data. SFL and YZH designed the study, supervised the experiments and gave the final approval of the manuscript. All authors read and approved the final manuscript.

## ETHICS APPROVAL AND CONSENT TO PARTICIPATE

All animal studies were approved by the Institutional Animal Care and Use Committee of Fudan University, Shanghai.

## Supporting information


Appendix S1

## Data Availability

The raw data supporting the conclusions of this manuscript will be made available by the authors, without undue reservation, to any qualified researcher.
