# Supplementary material for: Dnmt1 is required for the development of auditory organs via cell cycle arrest and Fgf signalling
Source: Cell Prolif. 2022 Mar 29;55(5):e13225. doi: 10.1111/cpr.13225 (PMC9136517; doi:10.1111/cpr.13225)
Supplement: Supplementary file 1 — Appendix S1 [file CPR-55-e13225-s001.docx]

**Dnmt1 is required in the development of auditory organs via cell cycle arrest and Fgf signaling**

Dongmei Tang^1#^, Shimei Zheng^2#^, Zhiwei Zheng^1#^, Chang Liu^1^, Jiner Zhang^2^, Renchun Yan^2^, Cheng Wu^2^, Na Zuo^2^, Lijuan Wu^2^, Hongfei Xu^3^, Shaofeng Liu^2^*, Yingzi He^1^*

1 ENT Institute and Otorhinolaryngology Department, Eye and ENT Hospital, State Key Laboratory of Medical Neurobiology, NHC Key Laboratory of Hearing Medicine Research, Fudan University, Shanghai, 200032, PR China

2 Department of Otolaryngology-Head and Neck Surgery, Yijishan Hospital of Wannan Medical College, Wuhu, Anhui, 241001, China

3 Department of Forensic Medicine, Soochow University, Suzhou, 215006, China

# Dongmei Tang, Shimei Zheng, Zhiwei Zheng contributed equally to this work.

***To whom correspondence should be addressed:**

Shaofeng Liu, PhD

Department of Otolaryngology-Head and Neck Surgery, Yijishan Hospital of Wannan Medical College

2 Zheshanwest Road, Wuhu, Anhui, 241001, China

E-mail: liusf_cn@163.com

Yingzi He, PhD

ENT institute and Otorhinolaryngology Department of Affiliated Eye and ENT Hospital, Fudan University

83 Fenyang Road, Shanghai, 200031, China

E-mail: [yingzihe09611@126.com](mailto:yingzihe09611@126.com)

| Gene Name | Forward Primer | Reverse Primer |
| --- | --- | --- |
| *Dnmt1* | 5'-ctggctcctggttcagactg-3' | 5'-gtgcttcaattttacagaatgatggc-3' |
| *eya1* | 5'-tagcacagacggattccacg-3' | 5'-tggtgctatcagaacgcaga-3' |
| *fgf3* | 5'-tgagcttcttggatccgagt-3' | 5'-tgccgctgactctctctaag-3' |
| *fgf8* | 5'-gacacatttgggagtcgagt-3' | 5'-aggtcagtactaaaagtcagggg-3' |
| *fgf10* | 5'-ctgctgcttctgttcctgtg-3' | 5'-agtgccttcttctccaaatgg-3' |
| *pea3* | 5'-agtgtgtttcgtgaaggtgc-3' | 5'-atacaagaggatggggtggg-3 |
| *lef1* | 5'-tcctctgggttggttctcac-3' | 5'-aaaagacgtccgctttcctc-3' |
| *cxcr4b* | 5'-tctgcgagcttacttacccg-3' | 5'-tgcacacactaactcgtcag-3' |
| *cxcr7b* | 5'-cccagtggaaagcatgaagg-3' | 5'-ccctgttgacctctgagctt-3' |
| *cxcr12* | 5'-tcgtagtagtcgctctgatgg-3' | 5'-ggacgcatatttggcaggtt-3' |
| *pax2* | 5'-ttccccacgtcagaacacat-3' | 5'-ccatgaaagtgaagatggcgt-3' |
| *pax5* | 5'-ttgggcattagttcacctgc-3' | 5'-ttcctgctcctcgtgtgg-3' |
| *tp53* | 5'-tcttttgaggtgcgtgtgtg-3' | 5'-acatgtatcgcagttcccca-3' |
| *cdkn1a* | 5'-acaagcggatcctacgttca-3' | 5'-ctacgagacgaatgcagctc-3' |

Gene Primer:


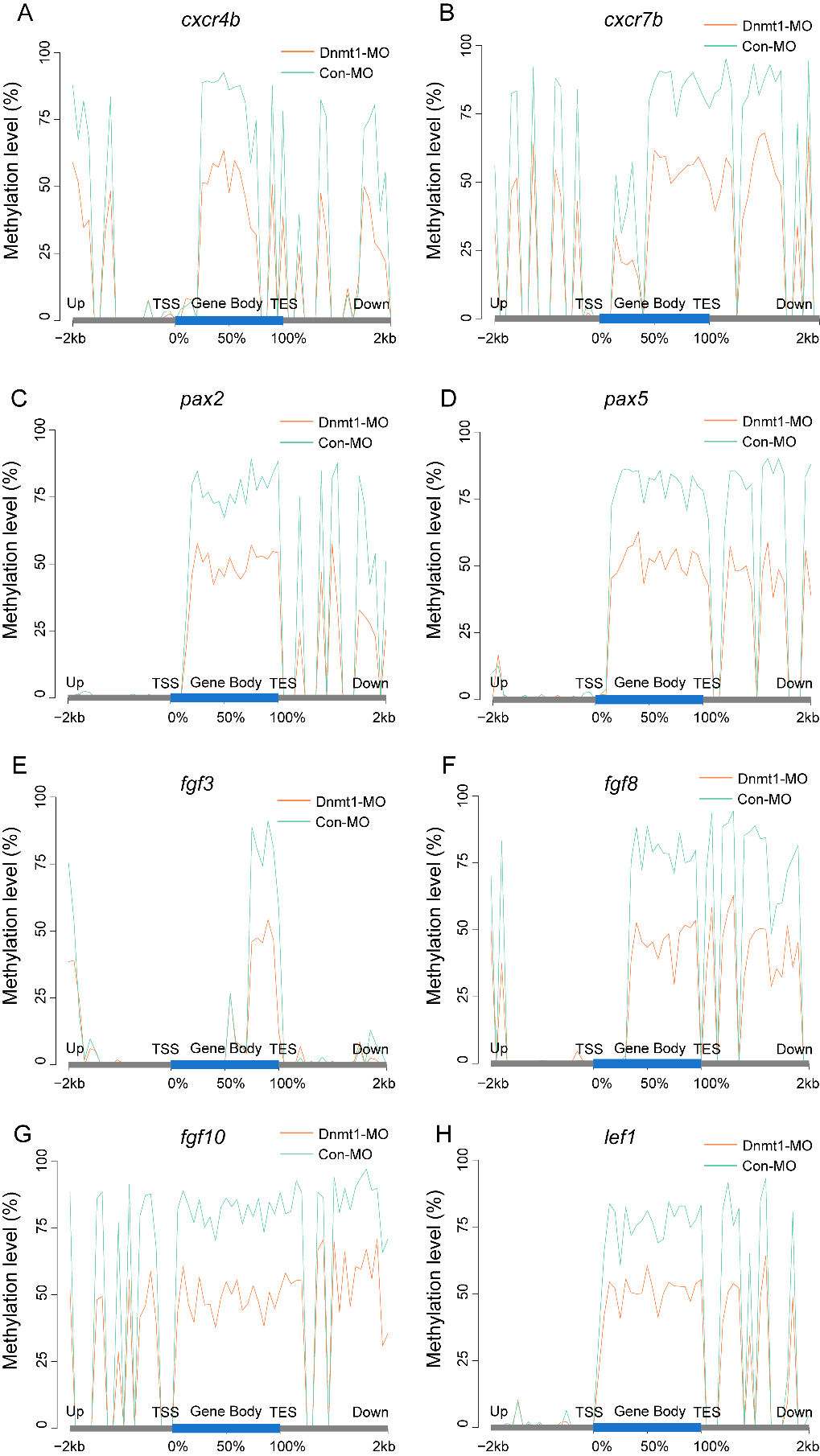


**Figure S1. Methylation levels in functional region of involved genes.** The methylation levels of *cxcr4b*, *cxcr7b*, *pax2*, *pax5*, *fgf3*, *fgf8*, *fgf10* and *lef1* in the functional region are significantly reduced in Dnmt1-MO morphants compared to the Con-MOs. TSS means transcription start site, and TES means transcription end site.
